# Effects of Dietary Supplementation of Coenzyme Q10 on Growth Performance, Biochemical and Physiological Attributes of Rainbow Trout (*Oncorhynchus mykiss*)

**DOI:** 10.1002/vms3.70324

**Published:** 2025-04-07

**Authors:** Hajar Asgari, Sayed Mohammad Ali Jalali, Mostafa Faghani, Azar Hematzadeh

**Affiliations:** ^1^ Department of Animal Sciences Sh K.C., Islamic Azad University Shahr‐e Kord Iran; ^2^ Research Center of Nutrition and Organic Products (RCNOP), Shahrekord Branch Islamic Azad University Shahrekord Iran

**Keywords:** blood attributes, body composition, coenzyme Q10, feed conversion ratio, rainbow trout

## Abstract

**Background:**

Coenzyme Q10 (CoQ10) is a natural antioxidant and plays a vital role in the energy production of animal cells; however, its physiological and biochemical properties in fish are unclear.

**Objectives:**

The current experiment was investigated to explore the effects of dietary CoQ10 on growth performance and biochemical and physiological attributes of rainbow trout (*Oncorhynchus mykiss*).

**Methods:**

A 56‐day feeding trial was conducted with 5 experimental diets supplemented with CoQ10 concentrations at 0, 25, 50, 100 and 200 mg kg^−1^ of diet and fed to 400 rainbow trout (10 ± 0.1 g, initial body weight).

**Results:**

Dietary supplementation with CoQ10, especially at the highest dietary level, significantly improved the feed conversion ratio, final body weight and lipid and protein efficiency ratio of fish (*p* < 0.05). Moreover, the fish carcass protein and lipid content significantly increased with supplementation of 200 mg CoQ10 kg^−1^ diet (*p* < 0.05). The blood serum levels of triglycerides (TGs), cholesterol (CHO) and uric acid significantly reduced, whereas albumin, total protein, high‐density lipoprotein and lymphocyte count increased with supplementation of 200 mg CoQ10 kg^−1^ diet (*p* < 0.05). Administration of 100 mg CoQ10 kg^−1^ diet significantly reduced the liver gene expression of sterol regulatory element‐binding protein1 (SREBP1), whereas supplementation with 200 mg CoQ10 kg^−1^ diet increased muscle gene expression of SREBP1 (*p* < 0.05). However, there were negative associations and correlations between blood TGs and CHO levels with liver and muscle SREBP1 gene expression.

**Conclusions:**

Overall, dietary supplementation of CoQ10, particularly at the levels of 100–200 mg kg^−1^ of diet, can improve growth performance and health in rainbow trout by modifying blood metabolites and SREBP1 gene expression.

## Graphical abstract

Coenzyme Q10 plays a crucial role in the electron transport chain and ATP production in the inner mitochondrial membranes. As a feed additive, it improves feed conversion ratio, specific growth rate and carcass crude protein while reducing the hepatosomatic index in rainbow trout. These effects are achieved by altering blood serum biochemical parameters, including high‐density lipoprotein (HDL), albumin and uric acid, as well as the gene expression of sterol regulatory element‐binding protein 1 (SREBP1) in the liver and muscle.

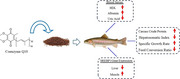

## 1 Introduction

Nowadays, the increased intensity of aquaculture and the use of high‐energy feeds make fish more susceptible to oxidative stress (Cardoso et al. 2010), which occurs when there is an imbalance between the production of free radicals and the accessibility of antioxidants in the fish body (Hoseinifar et al. 2020). Additionally, the oxidation of dietary organic material, such as carbohydrates, fatty acids and amino acids in animal tissues, provides the most load on the electron transport chain to adenosine triphosphate (ATP) production (Varela‐López et al. 2016). To combat these problems, natural components and feed additives such as probiotics, prebiotics, essential oils and medicinal herbs have gained attention and are being applied as eco‐friendly alternatives to synthetic materials and chemical anti‐stress agents in fish nutrition (Capkin et al. 2017).

Coenzyme Q10 (CoQ10) is a hydrophobic compound (Nepal et al. 2010) with a structure similar to that of vitamins E and K (Terčič et al. 2011) and naturally occurs in the inner membranes of mitochondria. Furthermore, CoQ10 has a critical role in the electron transport chain and ATP production (Lenaz et al. 2007; Shukla and Dubey 2018). The reduced form of CoQ10 (ubiquinol) exhibits high potency as an antioxidant agent against free radicals, protecting against oxidative risks, aiding in the regeneration of vitamin E and maintaining energy levels (Linnane et al. 2007; Martin et al. 2007). The CoQ10 has been reported to improve human and animal health against many diseases, such as diabetes, obesity, muscular dystrophy, heart disease, periodontal disease, cancer, Alzheimer's and ageing, accompanied by its anti‐inflammatory properties (Schmelzer et al. 2009; Sohet et al. 2009).

Although animals can synthesize CoQ10 in many tissues, including the liver and brain (Varela‐López et al. 2016), external sources are  required to obtain its beneficial properties due to the insufficient quantities synthesized naturally (Hernández‐Camacho et al. 2018). On the other hand, it is a low‐cost dietary additive with a broad range of beneficial effects (Feher et al. 2007; Shukla and Dubey 2018) that is well known in humans (Varela‐López et al. 2016) and poultry (Honda et al. 2010; Honda et al. 2013; Terčič et al. 2011; Jang and Moon 2016; Raeisi‐Zeydabad et al. 2017; Omidizadeh et al. 2021). Research conducted by El Basuini et al. (2020) demonstrated that supplementing CoQ10 in the diet of Nile tilapia (*Oreochromis niloticus*) at concentrations exceeding 20 mg kg^−1^enhanced health and growth performance. Another study observed the beneficial interaction effects of CoQ10 and vitamin C on growth performance, digestive enzyme activity, biochemical blood indices, and antioxidative capacity in Nile tilapia (El Basuini et al. 2021). Similarly, dietary supplementation of CoQ10 improved the growth performance of European seabass (*Dicentrarchus labrax L*.) larvae, as evidenced by increased weight gain, feed conversion ratio (FCR), specific growth rate (SGR), protein efficiency ratio (PER) and overall well‐being (El Basuini et al. 2022). Furthermore, treating fertile eggs of red tilapia (*Oreochromis aureus × Oreochromis mossambicus*) with CoQ10 resulted in improved growth, gut structure and antioxidant efficiency in the produced larvae. However, dietary supplementation with various antioxidant compounds (including vitamin E, CoQ10 [at 10 and 20 mg kg^−1^ diet] and selenium) showed that CoQ10 did not alter growth performance and blood biochemical indices in rainbow trout (Aramli et al. 2021). Moreover, dietary supplementation of CoQ10 in poultry diets improved the FCR (Geng et al. 2004; Gopi et al. 2014; Fathi 2015), mRNA level of sterol regulatory element‐binding protein 1 (SREBP1) in the liver (Jang and Moon 2016) and enhanced body weight gain and haematocrit (Geng et al. 2004; Huang et al. 2011). However, the effects of CoQ10 as a fish feed additive on the physiology and nutrition of rainbow trout (*Oncorhynchus mykiss*) remain largely unexplored. Specifically, the impact of CoQ10 on the gene expression of SREBP1 in the liver and muscle of rainbow trout has not been extensively investigated. SREBP1 is a crucial transcription factor that regulates lipid homeostasis by activating various lipogenic enzymes including  ATP citrate lyase, acetyl‐CoA carboxylase and fatty acid synthase. It also plays critical roles in the biosynthesis of phospholipids, fatty acids and triglycerides (TGs), which are essential for membrane synthesis and energy storage (Ruiz et al. 2020). Therefore, evaluating SREBP1 in liver and muscle tissues (the major sites of lipogenesis, lipolysis, and lipid deposition) as a key transcription factor regulating many genes, along with the biochemical parameters of blood serum, could provide a comprehensive perspective on the role of CoQ10 in lipid metabolism.

The present study aimed to investigate the potential effects of dietary supplemental CoQ10 on the growth performance and haematological attributes of rainbow trout (*O. mykiss*). In addition, the SREBP1 gene expression, which plays an integral role in regulating de novo lipogenesis, was evaluated in the liver and muscle tissues of the fish. Furthermore, correlations between SREBP1 gene expression and blood biochemical attributes were calculated to expolre potential associations.

## 2 Materials and Methods

### 2.1 Fish, Diets and Experimental Design

Four hundred rainbow trout fingerlings (initial body weight = 10 ± 0.1 g) were randomly distributed into 20 fibreglass tanks (20 fish/tank; 100 L) and fed with 5 experimental diets for 8 weeks. Each of the four tanks was randomly assigned to one of the experimental diets. To prepare the experimental diets, CoQ10 (98% purity; Shaanxi Jiahe Phytochchem Co. Ltd.) was added to commercial extruded rainbow trout feed without oil (3.0 mm diameter; FFT1 of Faradaneh Company) at the level of 0 (control), 25, 50, 100 and 200 mg kg^−1^ diet. As limited data on optimal dosages of CoQ10 in salmonid fish exist, a relatively wide range of concentrations was considered to evaluate its potential physiological and nutritional effects. The experimental diets were prepared at the beginning of the experiment by adding a mixture of fish oil, starch and CoQ10 to the basal diet. In the various experimental diets, the amount of CoQ10 was replaced by an equivalent amount of starch. All experimental diets were stored in a refrigerator at 5°C to maintain their quality during the experiment. The CoQ10 content in both the basal diet and the fish oil was considered negligible due to its instability during the extrusion process used in basal diet production, where the extruder and die temperatures were 130°C and 150°C (Alkoei et al. 2023), respectively. Additionally, the levels of CoQ10 in fish oil were considered low (Semeniuc et al. 2023), and fish oil was included at an equal concentration of 8% in all experimental diets (Table 1). The ingredients and chemical composition of the control diet are presented in Table 1. Fish were adapted to the experimental conditions for 2 weeks before starting the experiment and were fed the control diets. Feeding was conducted four times daily (08:00, 11:00, 14:00 and 17:00) at 2.5% body weight (Webster and Lim 2002), which was readjusted every 2 weeks based on the total biomass in the tanks. Water quality parameters were monitored daily during the experimental period and were as follows: T°C = 12 ± 2 (Thermometer), pH = 7.6 ± 0.2 (Portable digital pH meter Martini Instruments Model 201/digital), dissolved oxygen, 10.1 ± 0.3 mg L^−1^ (Waterproof Portable Dissolved Oxygen and BOD Meter model Hanna waterproof IP67) and total ammonia‐nitrogen = 0.2 ± 0.06 mg L^−1^ (Colorimetrically: Spectronic 601, Milton Roy Company, San Diego, CA, USA).

**TABLE 1:** Ingredients and proximate composition of control diet.

Ingredients	Amount (g/kg)
Fish meal (72% CP)	560
Soybean meal (48% CP)	40
Corn gluten meal (70% CP)	50
Starch^a^	240
Fish oil	80
Premix and additives^b^	30
Proximate composition (%)	
Moisture	8.5
Crude protein	46.9
Crude lipid	14.7
Crude fibre	0.8

^a^ Different experimental diet prepared with removing the equal amount of starch based on the dietary supplementation of coenzyme Q10 (25, 50, 100 and 200 mg/kg).

^b^ Includes vitamin, mineral and Immunoplus Faradaneh rainbow trout premix (26 g/kg), Prebiotic: Mannan‐oligosaccharide, (Bio‐Mos; Alltech, USA) (1 g/kg) and choline chloride (3 g/kg).

### 2.2 Growth Performance and Carcass Chemical Analysis

At the end of the experiment, all fish were euthanized using tricaine methanesulfonate (100 mg L^−1^) after a 24 h fast, and body weight and standard length were measured. Carcass chemical parameters (excluding internal organs; ground with a mincer) such as crude lipid (CL, Soxhlet system using petroleum ether) and crude protein (CP, Kjeldahl method) were measured according to AOAC procedures (AOAC 2005) for three fish per tank.

The following formulae were used to calculate the growth parameters (Jalali Haji‐Abadi et al. 2010; Jalali et al. 2021).

Condition factor (*K*)% = 100 × (*W*/*L* ^3^), where *W* indicates body weight (g) and *L* indicates body standard length (cm); weight gain (g) = final weight (g) − initial weight (g); FCR = feed intake (g)/weight gain (g); lipid efficiency ratio (LER)% = 100 × weight gain (g)/lipid intake (g); PER% = 100 × weight gain (g)/protein intake (g); hepatosomatic index (HSI)% = 100 × (liver weight (g)/body weight (g)); survival rate (SR)% = 100 × (final no. of fish in the tank/initial no. of fish in the tank); SGR% = 100 × (Ln*W*2 − Ln*W*1)/*T*, where *W*1 is the initial weight and *W*2 is the final weight of the fish, and *T* indicates the feeding period (56 days).

### 2.3 Blood Biochemical and Haematological Attributes

Three fish from each tank were randomly selected, and blood was collected from the caudal vein of the fish using a 2 mL syringe. One part was transferred to heparinized tubes for haematology and cell count parameters evaluation, and the other part was transferred to the tubes for blood serum separation (two times centrifuging at 5000 rpm for 5 min) to investigate biochemical attributes. The sera were frozen at −20°C until analysing blood biochemical attributes. Total cholesterol (CHO), TG, high‐density lipoprotein (HDL), albumin (ALB), total protein (TP) and uric acid (UA) in the serum were measured by colorimetrical methods (Pars Azmoon, diagnostic kits) and an Autoanalyzer machine (Persige 241—China) as described by Jalali et al. (2019) and Abedpour et al. (2017). The blood sample containing anticoagulant was used to estimate haematology indices, including haematocrit (PCV = packed cell volume), red blood cell count (RBC) and white blood cell count (WBC), white blood cell differential count, haemoglobin concentration (Hb), phagocytosis (PH) and the number of phagocytosis germs (Germ). The standard cyanomethemoglobin method was used to estimate HB. PCV was evaluated using the standard PCV method (Feldman et al. 2000). The total RBC was determined using a haemocytometer (Neubar) with manual counting, and WBCs were performed using the direct method (Jha et al. 2007). The erythrocyte indices, such as mean corpuscular volume (MCV), mean corpuscular haemoglobin (MCH) and mean corpuscular haemoglobin concentration (MCHC), were calculated as follows (Rodak et al. 2013):

$$\begin{array}{ccc} \mathrm{MCV} & = & \left(\mathrm{PCV}\times 10\right)/\mathrm{RBC};\mathrm{MCH}=\left(\mathrm{Hb}\times 10\right)/\mathrm{RBC}\\ \mathrm{MCHC} & = & \left(\mathrm{Hb}\times 100\right)/\mathrm{PCV} \end{array}$$

### 2.4 RNA Isolation and Real‐Time Polymerase Chain Reaction

Muscle and liver samples were taken from three fish of each tank to evaluate gene expression. A slice of liver/muscle (about 30 mg) was transferred to 1.5 cc Eppendorf tubes containing RNA*later* buffer (Nanozist Sanat Ahoora Co. Iran) incubated overnight at 4°C and then stored at −20°C until RNA extraction. Total RNA was isolated from liver and muscle samples using Trizol reagent (Sina Clone, Iran) according to the manufacturer's instructions, followed by DNase treatment. RNA concentration and purity were determined using a NanoDrop spectrophotometer (Thermo Ltd., USA) at 260 nm and 260 nm/280 nm ratio. First‐strand cDNA synthesis was performed using 500 ng of total RNA preparations (Jalali et al. 2011). The reverse transcription was accomplished by PrimeScript RT reagent Kit applying the manufacturer's instructions (Takara Ltd., Japan). For the removal of DNA, a DNase, RNase‐free kit (SINAclone, Iran) was used. Then, the reverse transcription products were stored at −20°C for qPCR.

Amplification and detection of specific products were performed using Real Time PCR machine (Rotor‐Gene 6000, Qiagen Inc. USA), SYBR Green I PCR Master Mix kit (Life Technologies, Carlsbad, California, USA) and specific primers. The primer sequences for the qPCR reactions are presented in Table 2. Designing the primers for SREBP1 and β‐actin was done using Fast PCR software. The β‐actin as the reference gene was used to estimate the relative expression of the studied gene. In this study, β‐actin was used as a non‐regulated and stably expressed reference gene. The stability of β‐actin was analysed by the Δ*CT* method (Schmittgen and Livak 2008). The kit SYBR Premix Ex Taq ɪɪ (Takara Ltd., Japan) was used for the qPCR reaction. The reaction chemicals were mixed on the ice according to Table S1 in 0.2 mL tubes and then placed in the Real Time PCR machine. The qPCR was performed according to the temperature setup brought in Table S2. The relative SREBP1 gene expression was quantified using equation 2^−(Δ^ *^CT^* ^sample−Δ^ *^CT^* ^control)^ method (Livak and Schmittgen 2001):

$$\mathrm{Relative}\;\mathrm{fold}\;\mathrm{change}\;\mathrm{in}\;\mathrm{gene}\;\mathrm{expression}=2^{-\Delta \Delta CT}$$

ΔCT=CTtargetgene−CTreferencegene

ΔΔCT=ΔCTtestsample−ΔCTControlsample

**TABLE 2:** Forward (F) and reverse (R) sequences of primers used in semi‐quantitative RT–PCR.

Gene	Primer name	Sequence (5′–3′)	Product length (pb)
SREBP1	KP342261.1	F: 5′‐CAAGCTGCCCATCAACCGTA‐3′ R: 5′‐GGCCACCAGGTCTTTAAGCTC‐3′	148
β‐actin	NM001124235	F: 5′‐TCCTTCCTCGGTATGGAGTCT‐3′ R: 5′‐TTACGGATGTCCACGTCACAC‐3′	80

### 2.5 Statistical Analysis

Data for recorded traits were analysed using analysis of variance procedures appropriate for a completely randomized design using the general linear model procedure of SAS 9.2 (SAS Institute Inc., Cary, NC, USA). Significant differences among treatment means were identified by using Tukey's multiple range test at *p* ≤ 0.05, unless otherwise stated. Specific orthogonal contrasts were used to test linear and quadratic effects of treatments. The principal component analysis (PCA) using biplots was applied to describe multivariate relationships among blood biochemical attributes and liver and muscle SREBP1 gene expression of fish fed with different experimental diets. Prior to analysis, the data were normalized and subsequently analysed using the PCA procedure of PRIMER software (PRIMER, Plymouth Routines in Multivariate Ecological Research; PRIMER‐E Ltd., version 6.1.15, Ivybridge, UK). Pearson correlation was performed to determine any correlation between SREBP1 gene expression and blood biochemical attributes (Minitab 16 Statistical Software, State College, PA, USA).

## 3 Results

### 3.1 Growth Performance and Carcass Chemical Composition

The effects of different dietary levels of CoQ10 supplementation on fish growth performance are presented in Table 3. Results showed that CoQ10 significantly reduced FCR, *K* and HSI, with the lowest values observed in fish fed diet containing 200 mg CoQ10 kg^−1^ (*p* < 0.05). However, the LER, PER, SGR, GAIN and final body weight (FBW) significantly increased with the inclusion of 200 mg CoQ10 kg^−1^ in the diet of rainbow trout (*p* < 0.05). Quadratic and linear regression analysis of different growth parameters showed almost same *R* ^2^ and probability value, except for LER and HSI, which were better described by quadratic equations (Table 3). Dietary supplementation of CoQ10 significantly altered the carcass content of CL and CP (*p* < 0.05, Table 4). The highest percentage of CP and CL were observed in fish fed with 200 mg CoQ10 kg^−1^ diet (*p* < 0.05).

**TABLE 3:** Effects of dietary supplementation with different levels of coenzyme Q10 on growth performance of rainbow trout (*Oncorhynchus mykiss*).

CoQ10	IBW	FBW	Gain	FI	FCR	PER	LER	SGR	K	HSI	SR
mg kg^−1^	g	g	g/fish	g/fish		%	%	%	%	%	%
**0**	10.73	42.78^c^	32.04^c^	38.97	1.22^a^	1.89^b^	5.35^b^	2.47^b^	1.03^a^	1.17^a^	98.33
**25**	10.63	42.63^c^	32.01^c^	38.72	1.21^ab^	1.90^b^	5.36^b^	2.48^b^	1.02^a^	1.07^b^	100.00
**50**	10.94	44.65^ab^	33.71^ab^	40.35	1.20^ab^	1.92^b^	5.42^b^	2.51^ab^	1.03^a^	0.94^c^	96.67
**100**	10.64	43.86^bc^	33.22^bc^	39.37	1.18^b^	1.94^b^	5.48^b^	2.53^ab^	0.94^b^	0.97^c^	100.00
**200**	10.87	45.71^a^	34.87^a^	39.91	1.15^c^	2.01^a^	6.28^a^	2.57^a^	0.88^c^	0.88^d^	100.00
***SP*** ^1^	*0.23*	*1.24*	*1.25*	*0.98*	*0.02*	*0.03*	*0.09*	*0.06*	*0.02*	*0.03*	*2.89*
***p* value**	*Ns*	^**^	^**^	*Ns*	^***^	^***^	^***^	^*^	^***^	^***^	*Ns*
**Linear; *R* ^2^**	0.041	0.475	0.478	0.107	0.758	0.775	0.851	0.397	0.858	0.681	0.058
***p* value**	*Ns*	^**^	^**^	*Ns*	^***^	^***^	^***^	^*^	^***^	^**^	*Ns*
**Quadratic; *R* ^2^**	0.051	0.477	0.477	*0.137*	*0.759*	*0.778*	*0.9635*	*0.411*	*0.859*	*0.814*	*0.059*
***p* value**	*Ns*	^**^	^**^	*Ns*	^***^	^***^	^***^	^*^	^***^	^***^	*Ns*

*Note*: Columns values with same superscript or no superscript are not significantly different (*p* < 0.05).

Abbreviations: FBW, final body weight; FCR, feed conversion ratio; FI, feed intake; HSI, hepatosomatic index; IBW, initial body weight; K, condition factor; LER, lipid efficiency ratio; Ns, not significant; PER, protein efficiency ratio; SGR, specific growth rate; SR, survival rate.

^1^ SP: pooled standard error.

* *p* < 0.05.

** *p* < 0.01.

*** *p* < 0.001.

**TABLE 4:** Effects of dietary supplementation with different levels of coenzyme Q10 on blood biochemical attributes and carcass crude protein (CP) and crude lipid (CL) of rainbow trout *(Oncorhynchus mykiss)*.

CoQ10	ALB	TP	TG	CHO	UA	HDL	CP	CL
mg kg^−1^	g/dL	g/dL	mg/dL	mg/dL	mg/dL	mg/dL	% as is	% as is
**0**	1.40^c^	2.55^c^	111.63^c^	134.97^d^	2.17^a^	33.01^c^	15.64^b^	4.47^d^
**25**	1.80^bc^	3.67^b^	149.10^b^	166.05^b^	1.74^b^	33.53^c^	15.52^b^	4.62^c^
**50**	1.86^b^	2.80^c^	156.91^a^	152.15^c^	1.07^c^	60.84^a^	15.29^b^	4.93^a^
**100**	2.19^b^	4.07^a^	146.60^b^	211.45^a^	0.98^c^	63.60^a^	16.18^a^	4.76^b^
**200**	2.60^a^	4.32^a^	104.45^d^	58.90^e^	1.89^b^	54.44^b^	16.40^a^	4.95^a^
***SP*** ^1^	*0.22*	*0.14*	*1.90*	*5.40*	*0.12*	*2.86*	*0.2091*	*0.0423*
***p* value**	^***^	^***^	^***^	^***^	^***^	^***^	^***^	^***^
**Linear; *R* ^2^**	0.819	0.628	0.159	0.293	0.008	0.329	0.622	0.511
***p* value**	^***^	^***^	*Ns*	^*^	*Ns*	^*^	^***^	^**^
**Quadratic; *R* ^2^**	0.848	0.668	0.841	0.876	0.924	0.799	0.627	0.644
***p* value**	^***^	^***^	^***^	^***^	^***^	^***^	^***^	^***^

*Note*: Columns values with same superscript or no superscript are not significantly different (*p* < 0.05).

Abbreviations: ALB, albumin; CHO, cholesterol; CL, crude lipid. CP, crude protein; HDL, high‐density lipoprotein; Ns, not significant; TG, triglycerides; TP, total protein; UA, uric acid.

^1^ SP: pooled standard error.

* *p* < 0.05.

** *p* < 0.01.

*** *p* < 0.001.

### 3.2 Blood Biochemical and Haematological Attributes

The highest blood serum levels of ALB and TP and the lowest TG and CHO were found in fish fed with 200 mg CoQ10 kg^−1^ diet. Additionally, the lowest level of UA in fish serum was observed at 100 mg dietary supplemental CoQ10 kg^−1^. Regression analysis indicated that quadratic models provided a better fit than linear functions for the responses of TG, CHO, HDL and UA to dietary CoQ10 levels, as evidenced by higher *R* ^2^ values and lower *p* values (Table 4). All haematological attributes, except for lymphocyte count, were not significantly affected by different experimental diets (*p *> 0.05; Table 5). The highest lymphocyte count, expressed as a proportion of total white blood cells, was observed in fish fed 200 mg CoQ10 kg^−1^ diet (Table 3).

**TABLE 5:** Effects of dietary supplementation with different levels of coenzyme Q10 on haematological attributes of rainbow trout *(Oncorhynchus mykiss)*.

CoQ10	PCV	RBC	HGB	WBC	NUT	LYM	MON	EOS	BASO	MCV	MCH	MCHC
mg kg^−1^	%	×10⁶/µL	g/dL	/µL	%WBC	%WBC	%WBC	%WBC	%WBC	(fL)	pg/cell	g/dL
**0**	37.33	3.10	12.40	11383	20.33	72.0^cb^	5.00	2.00	0.67	120.8	40.13	33.22
**25**	39.33	3.33	13.07	13166	17.67	74.0^abc^	3.67	3.33	1.33	118.0	39.20	33.22
**50**	39.67	3.33	13.27	10467	21.67	71.0^c^	4.00	2.33	1.00	119.4	39.92	33.43
**100**	33.00	2.63	10.93	12167	18.67	76.3^ab^	3.67	2.00	0.67	126.2	41.80	33.13
**200**	32.67	2.63	10.93	12183	16.33	77.3^a^	3.67	2.00	0.67	131.6	44.17	33.53
***SP*** ^1^	*5.65*	*0.64*	*1.87*	*1026*	*2.63*	*2.39*	*1.26*	*0.97*	*0.68*	*12.3*	*4.26*	*0.19*
***p* value**	*Ns*	*Ns*	*Ns*	*0.08*	*0.18*	^*^	*Ns*	*Ns*	*Ns*	*Ns*	*Ns*	*Ns*
**Linear; *R* ^2^**	0.230	0.202	0.218	0.016	0.221	0.447	0.087	0.061	0.045	0.216	0.234	0.200
***p* value**	*Ns*	*Ns*	*Ns*	*Ns*	*Ns*	**	*Ns*	*Ns*	*Ns*	*Ns*	*Ns*	*Ns*
**Quadratic; *R* ^2^**	0.231	0.204	0.219	0.022	0.254	0.449	0.159	0.0624	0.050	0.219	0.239	0.265
***p* value**	*Ns*	*Ns*	*Ns*	*Ns*	*Ns*	**	*Ns*	*Ns*	*Ns*	*Ns*	*Ns*	*Ns*

*Note*: Columns values with same superscript or no superscript are not significantly different (P <0.05).

Abbreviations: BASO, basophil; EOS, eosinophil; HGB, haemoglobin; LYM, lymphocytes; MCH, mean corpuscular haemoglobin; MCHC, mean corpuscular haemoglobin concentration; MCV, mean corpuscular volume; MON, monocytes; Ns, not significant; NUT, neutrophil; PCV, packed cell volume; RBC, red blood cell; WBC, white blood cell count.

^1^ SP: pooled standard error.

* *p* < 0.05.

### 3.3 SREBP1 Gene Expression

**FIGURE 1:**
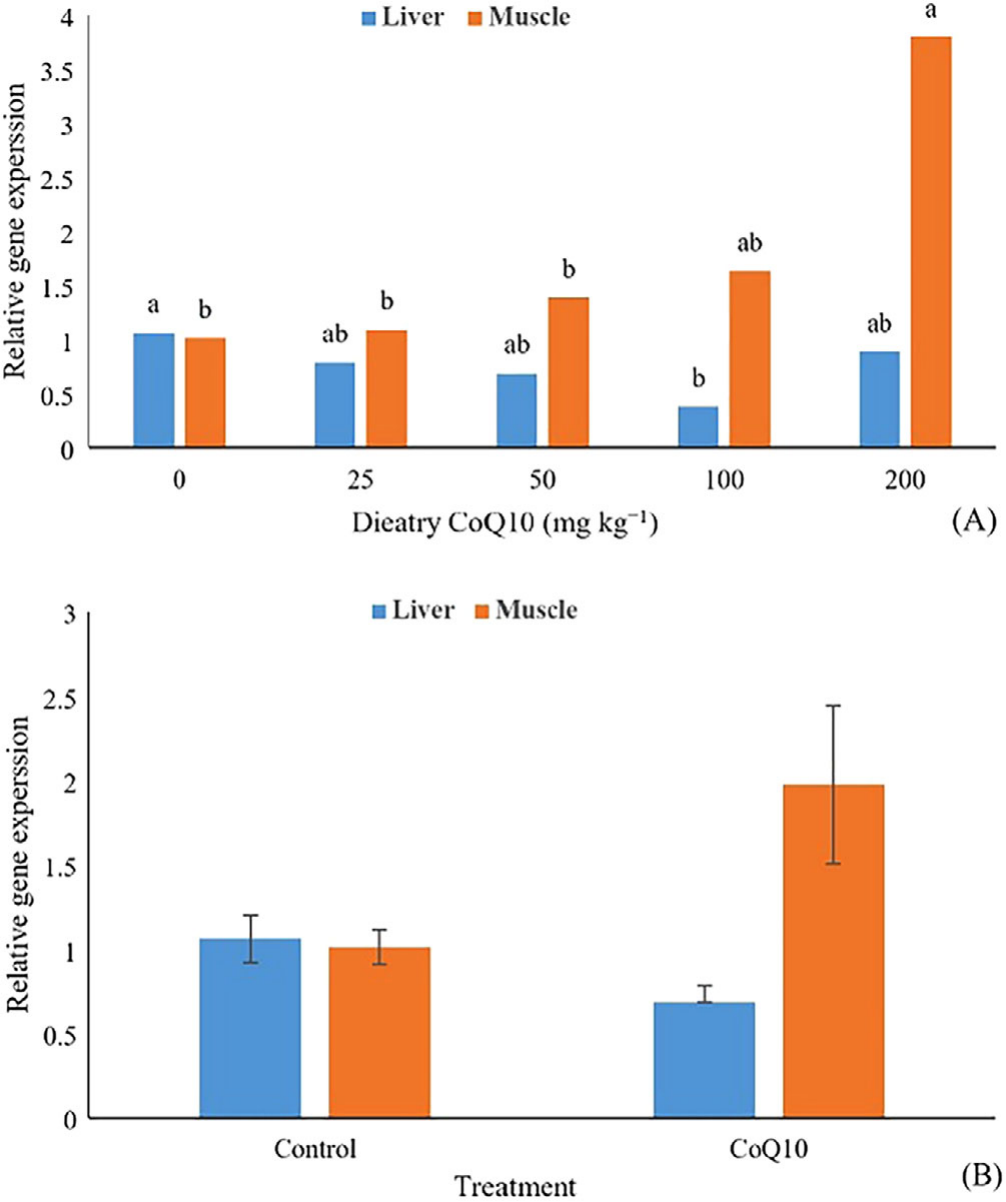
Effects of dietary supplementation with different levels of coenzyme Q10 (A) and independent comparison (B) on gene expression of sterol regulatory element‐binding protein 1 (SREBP1) in the liver and muscle of rainbow trout (*Oncorhynchus mykiss*).

Experimental diets affected the SREBP1 gene in the liver and muscle of fish. The lowest liver gene expression occurred in fish fed with 100 mg CoQ10 kg^−1^ diet, whereas the highest muscle gene expression was observed through dietary supplementation of 200 mg kg^−1^ CoQ10 (*p* < 0.05; Figure 1A). Independent comparisons exhibited a decrease in liver SREBP1 gene expression and a concurrent increase in muscle SREBP1 gene expression in fish fed diets containing CoQ10 (*p* < 0.05; Figure 1B).

### 3.4 Correlations

**FIGURE 2:**
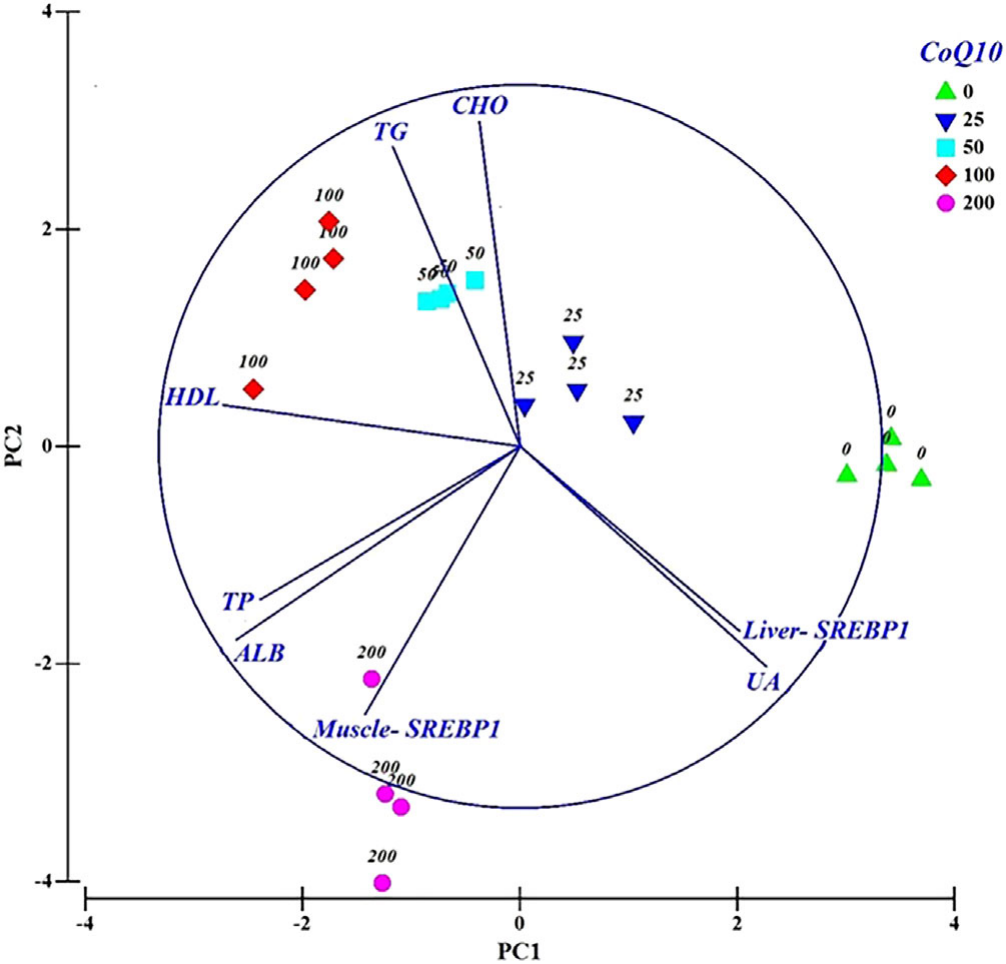
Principal component analysis (PCA) biplot of blood biochemical attributes and gene expression of SREBP1 in the liver and muscle of rainbow trout (*Oncorhynchus mykiss*) fed diets supplemented with varing levels of coenzyme Q10 (0, 25, 50.100 and 200 mg kg^−1^). The variation of PC1 and PC2 were 41.7 and 36.3%, respectively. ALB: albumin; CHO: cholesterol; HDL: high‐density lipoprotein; SREBP1: sterol regulatory element‐binding protein 1; TG: triglycerides; TP: total protein; UA: uric acid.

Multivariate analyses of blood biochemical attributes and SREBP1 gene expression of the liver and muscle are shown in Figure 2. The PCA revealed that the PC1 separated SREBP1 gene expression and experimental diets. Liver gene expression, along with control and 25 mg CoQ10 kg^−1^ diet located at the positive part of PC1, whereas muscle gene expression, accompanied with 50–200 mg CoQ10 kg^−1^ diet, is located in the negative part of PC1. Furthermore, liver SREBP1 gene expression was associated with the blood level of UA, whereas muscle SREBP1 gene expression was mostly associated with ALB and TP of blood serum, especially in fish fed the 200 mg CoQ10 kg^−1^ diet (Figure 2). The liver and muscle SREBP1 gene expression displayed a positive association (Figure 2) and correlation with UA and ALB in the serum of fish (*R* = 0.66; *p* < 0.001, Table 6), respectively. The CHO and TG showed moderate negative association and correlation with liver (*R* = −0.5; *p* < 0.05) and muscle (*R* = −0.42 and 0.57, *p* < 0.05) SREBP1 gene expression (Figure 2 and Table 6). However, UA levels in blood serum had a significant negative association and correlation with other lipid blood component factors, such as CHO, TG and HDL (*R* = −0.79, −0.77 and −0.62, respectively).

**TABLE 6:** Pearson correlation coefficients of SREBP1 gene expression and blood biochemical attributes of rainbow trout.

	ALB	TP	TG	CHO	UA	HDL	Liver SREBP1
**Liver‐SREBP1**	−0.218^Ns^	−0.265^Ns^	−0.498^*^	−0.504^*^	0.663^***^	−0.477^*^	—
**Muscle‐SREBP1**	0.662^***^	0.533^**^	−0.423^*^	−0.569^**^	0.127^Ns^	0.338^Ns^	0.166^Ns^

Abbreviations: ALB, albumin; CHO, cholesterol; HDL, high‐density lipoprotein; Ns, not significant; SREBP1, sterol regulatory element‐binding protein 1; TG, triglycerides; TP, total protein; UA, uric acid.

* *p* < 0.05.

** *p* < 0.01.

*** *p* < 0.001.

## 4 Discussion

In the current experiment, the growth performance of rainbow trout improved with dietary inclusion of more than 50 mg CoQ10 kg^−1^ diet, and FCR and PER improved only at 200 mg CoQ10 kg^−1^ diet. Interestingly, the improvement in growth performance was accompanied by a reduction in the condition factor (*K*) and HSI. The antioxidant properties of CoQ10 (Varela‐López et al. 2016) and its role in ATP production (Shukla and Dubey 2018) are the main reasons for the observed improvement in growth performance. Other roles such as helping re‐synthesis of vitamin E (Linnane et al. 2007; Martin et al. 2007) and increasing digestive enzyme activity (El Basuini et al. 2020), could also be considered contributing factors to the enhancement of growth performance. In this regard, El Basuini et al. (2020) recently tested different dietary supplementation levels of CoQ10 (0, 10, 20, 30 and 40 mg kg^−1^ diet) in Nile tilapia (*O. niloticus*) diet (12 g initial body weight). Their results exhibited that the inclusion of more than 20 mg CoQ10 kg^−1^ diet improved growth performance–related parameters of fish, including weight gain, SGR and feed efficiency. On the basis of results obtained in our study, a higher dietary supplementation of CoQ10 than that recommended for tilapia is required for the diet of rainbow trout (11 g initial body weight) owing to its higher protein and lipid content (NRC 2011). The crude protein and lipid levels in the current experimental diet were approximately 47% and 14.7%, respectively, which were about 1.6 and 1.8 times higher than those in the Nile tilapia tested diets (30% and 8%, respectively). Therefore, differences in feeding habits and nutrient requirements between tilapia and trout may be the main factors influencing their varying needs for CoQ10 dietary supplementation. Regarding the role of CoQ10 in ATP synthesis and production (Hemmin and Rajak 2006; Shukla and Dubey 2018), it may reduce the utilization of protein and lipids for energy supply. Therefore, CoQ10 can improve the protein and LER and enhancing protein and lipid retention in the whole body of fish. Consequently, the increased carcass fat and protein contents may be due to the effects of CoQ10 on energy production and subsequently its sparing effect on the efficiency of lipid/protein utilization (Kagan et al. 1996).

Blood attributes are considered accurate indicators of fish health status and their response to external conditions (El Basuini et al. 2016). Therefore, evaluating fish blood metabolites in response to dietary supplementation of CoQ10 can provide valuable criteria for assessing the efficiency of fish diet. The increasing serum levels of CHO and TG in fish fed up to 100 mg CoQ10 kg^−1^ diet and their reduction in fish fed 200 mg CoQ10 kg^−1^ diet might be the result of CoQ10's controlling effects on enzyme activities involved in their synthesis. It is worth noting that CoQ10 biosynthesis involves three main steps: the synthesis of benzoquinone from tyrosine, the synthesis of the isoprene side chain from acetyl‐CoA via the mevalonate (CHO‐synthetic) pathway and the condensation of the benzoquinone and mevalonate structures (Wu G. 2017). Thus, the biosynthetic reactions of CHO and CoQ10 are similar until the production of farnesyl pyrophosphate in the mevalonate pathway (Turunen et al. 2004; Cox and Nelson 2008). Supplementation of CoQ10 up to 100 mg kg^−1^ of diet may reduce the use of acetyl CoA (precursor of CHO and fatty acids) for ubiquinone synthesis. This range of CoQ10 may reciprocally shift acetyl CoA towards the synthesis of CHO and TG in the liver, thereby increasing their levels in the serum. Notably, some researchers have demonstrated that CoQ10 could inhibit liver production of CHO (Honda et al. 2010; 2013) by suppressing the enzyme activity of hydroxymethyl glutaryl CoA (HMG‐CoA) reductase (Wu G. 2017), a rate‐limiting enzyme in the CHO /CoQ10 synthetic pathway. This effect might have occurred in fish fed 200 mg CoQ10 kg^−1^, which requires further research in rainbow trout (*O. mykiss*). Raeisi‐Zeydabad et al. (2017) showed a reduction trend in blood CHO and TG concentrations in broiler chicks fed with diets containing CoQ10 (Raeisi‐Zeydabad et al. 2017). In another research, a trend of decreasing CHO levels in the serum and egg was reported in laying hens fed CoQ10 (Honda et al. 2013). Differences in the serum values of TG and CHO between fish and other vertebrates may be related to the various physiological responses. For instance, the recycling of lipids as TG and CHO forms in fish is typically higher (hyperlipidemic and hypercholesterolemic) than in birds and mammals due to the lack of efficient clearance of lipids in fish blood (Turchini et al. 2022). The increased blood concentrations of TP and ALB in response to dietary supplementation of CoQ10 were observed in this study. In contrast, Gopi et al. (2014) reported that serum TP and ALB of broiler chickens did not change with dietary supplementation of CoQ10 (Gopi et al. 2014). The increasing serum levels of TP and ALB could be considered a sign of innate immune system improvement accompanied by higher immunoglobulin levels (Hussein 1996). Therefore, it seems that the addition of CoQ10 to the diet of rainbow trout can improve the nutritional and immunological status of fish. The HDL is the most important lipoprotein in fish blood (Zheng et al. 2004) and performs the transport of CHO from tissues to the liver, whereas the blood ALB acts as the main fatty acid‐binding protein, transporting them through body tissues (Van der Vusse 2009). Hence, changes in the blood levels of these metabolites can be related to modifications in the metabolism process in the body, whereas increasing the level of the other blood proteins may express a protein‐sparing effect along with an improvement of energy production caused by CoQ10 consumption (Hemmin and Rajak 2006). The blood UA level of rainbow trout declined with dietary supplementation of CoQ10. This may be related to the enhancement of ATP production via the availability of CoQ10 for the electron transport chain and, consequently, an increase in the use of purines (e.g., adenine) in the synthesis of ATP, resulting in a decline in its degradation to UA (Berg et al. 2015). It is worth mentioning that despite the ability of fish to degrade UA, catabolism of UA and its precursors (purines) results in the generation of hydrogen peroxide that can damage erythrocytes and cell membranes if levels of antioxidants are inadequate (Berg et al. 2015). Therefore, a reduction in blood UA level may be a sign of the health of the fish. The regression analysis of TG, CHO and HDL showed concave quadratic function (TG = −0.0045*x* ^2^ + 0.7978*x* + 121.24; CHO = −0.0096*x* ^2^ + 1.5999*x* + 126.87; HDL = −0.0023 × 2 + 0.5866*x* + 29.702; *x* = CoQ10 dietary level) where the maximum blood levels of them were predicted by the derivation of equations and were 88.7, 83.3 and 127.5 mg CoQ10 kg^−1^diet, respectively. Blood UA showed a convex quadratic equation (UA = 0.0001*x* ^2^ – 0.0242*x* + 2.1703; *x* = CoQ10 dietary level) where the minimum was achieved at 121 mg CoQ10 kg^−1^diet. Therefore, it appears that the most significant influence of CoQ10 dietary level on blood attributes was observed around 83–128 mg CoQ10 kg^−1^diet. These ranges could be considered for future research on the feeding level of CoQ10 for rainbow trout.

The blood haematological attributes, except for lymphocytes, were not influenced by dietary supplementation of CoQ10. The highest lymphocytes count was observed in fish fed with 200 mg CoQ10 kg^−1^ diet (Table 3), which can be a sign of improved immunological condition in fish (Urbinati and Carneiro 2001). In this regard, pervious studies have also shown that dietary CoQ10 supplementation led to an increase in the lymphocyte count of broiler chickens (Sharifi et al. 2016; Raeisi‐Zeydabad et al. 2017) and humans (Turunen et al. 2004). In agreement with our findings, feeding CoQ10 did not affect the haematocrit, RBC and Hb (Asadi et al. 2013; Fathi 2015; Nemati et al. 2017; Raeisi‐Zeydabad et al. 2017) as well as the level of MCHC, MCV and MCH indices (Asadi et al. 2013).

In the current experiment, dietary supplementation of CoQ10 decreased the gene expression of SREBP1 in the liver while increasing the same gene in the muscles. The experimental diets affected the SREBP1 gene, with the lowest expression observed in the liver of fish fed 100 mg CoQ10 kg^−1^diet and the greatest expression in the muscle of fish fed 200 mg CoQ10 kg^−1^ diet. These findings are consistent with the results of Jang and Moon (2016), who reported a downregulated expression of SREBP1 in the liver of laying hens fed with a dietary administration of 100 mg CoQ10 kg^−1^. Previous studies have reported that CoQ10 can downregulate lipid synthesis gene expression (e.g., SREBP1, acetyl‐CoA carboxylase and fatty acid synthase) and upregulate the expression of fatty acid oxidation genes (e.g., peroxisome proliferator–activated receptors α and carnitine palmitoyltransferase‐1) in the liver of mice (Chen et al. 2019) and hen (Sharideh et al. 2020). Among the three isoforms of the SREBPs family, which are the primary regulator's genes for lipid and CHO synthesis (Eberlé et al. 2004; Honda et al. 2013), SREBP1 is capable of activating all SREBP‐responsive genes containing those included in the syntheses of CHO, fatty acids and TG (Zhu et al. 2020). The lower expression of the SREBP1 gene in the liver of fish fed CoQ10 (Figure 1A,B) may lead to a reduction in lipid synthesis in the liver, whereas its higher expression in the muscle may promote lipid biosynthesis in the muscle. This change is reflected in the HSI, which was reduced in fish fed CoQ10, and in the increased total crude lipid content in the whole carcass of these fish (Table 4). Research has shown that SREBP1 is a key factor in regulating lipid metabolism by activating lipogenic enzymes such as ATP citrate lyase, acetyl‐CoA carboxylase and fatty acid synthase. Additionally, SREBP1 plays a critical role in the biosynthesis of phospholipids, fatty acids and TGs, which are essential for membrane synthesis and energy storage (Ruiz et al. 2020). Therefore, our results suggest that feeding CoQ10 to rainbow trout affects the expression of the SREBP1 gene differently in the liver and muscle, overall improving liver function by reducing lipogenesis and HSI and enhancing fish growth by increasing whole body protein and lipid content. The negative correlation between SREBP1 gene expression and serum TG and CHO in fish indicates a relationship between this gene and neutral lipid metabolism. However, there is not enough data regarding the specific role of CoQ10 in the muscle tissue. Moreover, lipid metabolism pattern differ between the liver and muscle of fish, whereby the liver lipid contents in rainbow trout are much lower than the lipid contents of muscle (Turchini et al. 2022). The data resulting from this experiment suggest that higher dietary supplemental levels of CoQ10 (100–200 mg kg^−1^ diet) resulted in different metabolic effects on the SREBP1 gene expression in the liver and muscle of fish, although the underlying reason is unclear and requires further study. Additionally, the muscle SREBP1 gene expression was positively correlated with ALB, which acts as a plasma fatty acid transporter to muscle cells (Van der Vusse 2009), and SREBP1 may be related to fatty acid metabolism in fish muscle (Dong et al. 2017). The positive correlation between the serum UA/ALB and liver/muscle SREBP1 gene expression could be considered in future studies of the lipid and protein metabolism in the liver and muscle of fish.

## 5 Conclusion

Overall, the results showed that dietary supplementation with CoQ10, particulary at the levels of 100–200 mg kg^−1^ diet, improved the growth performance, FCR, LER and PER of rainbow trout. Additional, relatively high dietary levels of CoQ10 caused variation in the liver and muscle SREBP1 gene expression as well as blood metabolites in rainbow trout.

## Author Contributions

**Hajar Asgari**: investigation, conceptualization, resources, writing – original draft. **Sayed Mohammad Ali Jalali**: conceptualization, data curation, formal analysis, methodology, project administration, supervision, writing – review and editing. **Mostafa Faghani**: investigation, methodology. **Azar Hematzadeh**: conceptualization, validation.

## Ethics Statement

The protocol for fish handling, experimental methods, and all data collection during the trial were approved by the ethics committee of I.A.U. (Cod. Num. IR.IAU.SHK.REC.1399.004).

## Conflicts of Interest

The authors declare no conflicts of interest.

## Supporting information



Supporting Information

## Data Availability

The data that support the findings of this study are available from the corresponding author upon reasonable request.
