# Supplementary material for: Effects of Dietary Supplementation of Coenzyme Q10 on Growth Performance, Biochemical and Physiological Attributes of Rainbow Trout (Oncorhynchus mykiss)
Source: Vet Med Sci. 2025 Apr 7;11(3):e70324. doi: 10.1002/vms3.70324 (PMC11974040; doi:10.1002/vms3.70324)
Supplement: Supplementary file 1 — Supporting Information [file VMS3-11-e70324-s001.docx]

Supplementary Table 1. Chemicals used in Real-Time PCR reaction

| **Chemicals** | **Amount** | **Concentration** |
| --- | --- | --- |
| Master SYBR Green | 10 µl | - |
| Forward primer | 0.5 µl | 10 pmol |
| Reverse primer | 0.5 µl | 10 pmol |
| CDNA | 1 µl | 0.5 Mg |
| dH_2_O | 8 µl | - |

Supplementary Table 2. The temperature set up of Real-Time PCR reaction

| **Stages of Real-Time PCR** | **Temperature (˚C)** | **Reaction time (S)** | **Cycles No.** |
| --- | --- | --- | --- |
| Initial Denaturation | 95 | 240 | 1 |
| Denaturation | 94 | 5 | 40 |
| Primer annealing | 65-57 | 20 | 40 |
| Extension | 72 | 20 | 40 |
